# Building from the Bottom Up: A Closer Look into the Teaching and Learning of Life’s Principles in Biomimicry Design Thinking Courses

**DOI:** 10.3390/biomimetics7010025

**Published:** 2022-02-05

**Authors:** Laura Lee Stevens, Michelle Fehler, Deborah Bidwell, Asha Singhal, Dayna Baumeister

**Affiliations:** 1 Industrial Design Engineering, The Hague University of Applied Sciences, 2521 EN Den Haag, The Netherlands; 2 The Design School, College of Global Futures, Arizona State University, Tempe, AZ 85287, USA; mfehler@asu.edu (M.F.); dbaumeis@asu.edu (D.B.); 3 Department of Biology Charleston, College of Charleston, Charleston, SC 29424, USA; bidwelld@cofc.edu; 4 Hybrid Futures, 10249 Berlin, Germany; asha@hybridfutures.de

**Keywords:** biomimicry, life’s principles, pedagogy, design thinking, science education, biology, analogical thinking, innovation

## Abstract

Biomimicry education is grounded in a set of natural design principles common to every known lifeform on Earth. These Life’s Principles (LPs) (cc Biomimicry 3.8), provide guidelines for emulating sustainable strategies that are field-tested over nearly four billion years of evolution. This study evaluates an exercise for teaching LPs to interdisciplinary students at three universities, Arizona State University (ASU) in Phoenix, Arizona (USA), College of Charleston (CofC) in Charleston, South Carolina (USA) and The Hague University of Applied Sciences (THUAS) in The Hague (The Netherlands) during the spring 2021 semester. Students researched examples of both biological organisms and human designs exhibiting the LPs. We gauged the effectiveness of the exercise through a common rubric and a survey to discover ways to improve instruction and student understanding. Increased student success was found to be directly linked to introducing the LPs with illustrative examples, assigning an active search for examples as part of the exercise, and utilizing direct assessment feedback loops. Requiring students to highlight the specific terms of the LP sub-principles in each example is a suggested improvement to the instructions and rubric. An iterative, face-to-face, discussion-based teaching and learning approach helps overcome minor misunderstandings. Reiterating the LPs throughout the semester with opportunities for application will highlight the potential for incorporating LPs into students’ future sustainable design process.

## 1. Introduction

Biomimicry is an emerging discipline that looks towards nature to learn how to create resilient, regenerative and sustainable solutions to human challenges. We as humans, are relearning to both apply and teach these biological design lessons through the process of Biomimicry Design Thinking, a framework for translating biology to design. Biomimicry Design Thinking merges Biomimicry Thinking and Design Thinking, to examine the design challenge context, discover existing solutions in nature, create ideas and evaluate them to generate innovative design solutions [1]. Biomimicry practitioners ask the same question that many designers would also put forward, ‘what does the design need to do?’. However, when looking for solutions, instead of focusing on human design precedents, biomimicry practitioners begin by looking to nature to discover time tested solutions backed by more than 3.8 billion years of ‘research and development’. 

By looking at the natural function in context, and translating natural strategies and mechanisms to the design context, biomimicry practitioners practice analogical reasoning. This process of looking at one context (e.g., biology) and applying this to the second context (e.g., design) is called Analogical Thinking [2,3]. One might explore Analogical Thinking in biomimetic examples such as Sharklet’s anti-fouling surface texture that emulates the form of shark skin micro-pattern [4]; the life-friendly and non-toxic plywood that mimics the biochemical process that blue mussels use to create adhesives that can function under wet conditions [5] or innovative solutions for learning optimal paths for evacuation inspired by emulation of slime mold self-organization and learning without a brain [6]. The field of Biology inspired Design (BID) including Biomimicry Design Thinking has been gaining momentum, and educational programs such as those offered by the Biomimicry Institute and Biomimicry 3.8 have expanded rapidly around the globe. There are approximately 29 institutions worldwide who teach some form of biological translation for innovation, which include Biomimetics, Biomimicry, BID, and Bionics [7]. The Master of Science in Biomimicry at Arizona State University has spawned multiple cohorts since 2015 who have, in turn, initiated new learning programs, continuing the expansion of the practice. Other examples of programs include Biomimicry Commons in Canada, Biomimicry Academy in Berlin, Learn Biomimicry in South Africa, and universities with their own biomimicry programs. 

Up to now, research has been conducted on biomimicry and bio-inspired design didactics. Yen et al. [8] found that creativity increased through analogical reasoning liking functional biology to human design challenges. Yen et al. [9] synthesized their pedagogy and lessons learned, assessing their interdisciplinary bioinspired design course at Georgia Institute of Technology. They noted students were challenged to identify, understand, map, and translate biology through analogical thinking (abstracting design principles). They also noted that although students naturally make analogies between engineering and natural history, these analogies tend to be superficial. Both biology and engineering students struggled to explain why the natural models were good analogies. Nagel et al. [10] have been conducting meaningful research into exploring the infusion of biomimicry into engineering courses. This research is to promote a continuation of foundational biology knowledge, foster interdisciplinary thinking in problem solving and train students to keep a flexible and adaptable mind as the world changes. Rowland [11] wrote of the biomimicry step-by-step methodology, and Rovalo & McCardle [12] cited the difficulty of making the analogical transfer of the strategies and mechanisms from biology to design. Applying strategies from nature correctly through the translation of biology into design continues to be one of the most challenging steps in the biology inspired design realm [3,13,14,15,16]. However, more research on the effectiveness of ‘best practices’ in biomimicry and bio-inspired design is needed. 

**Figure 1:**
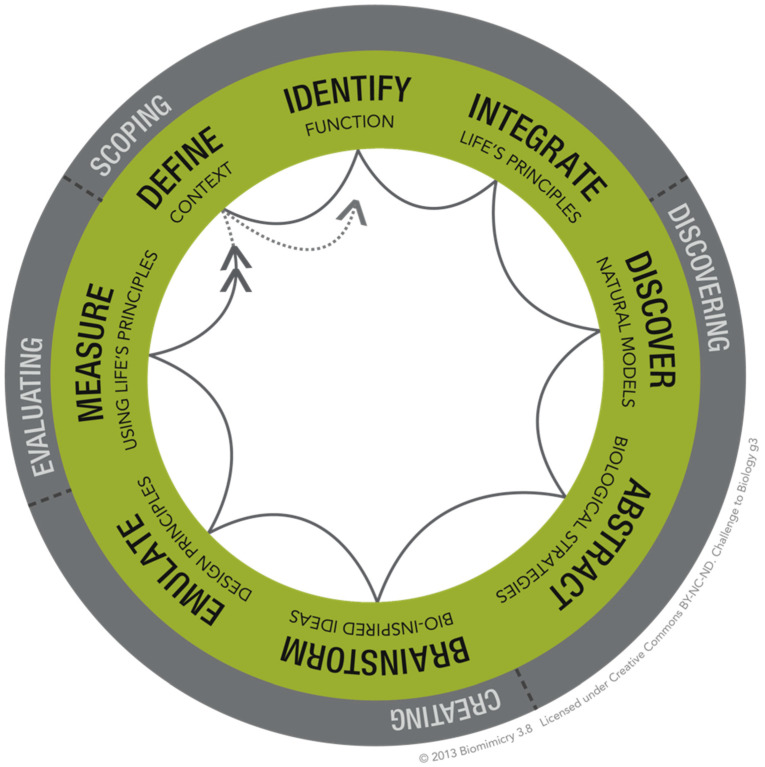
Biomimicry Thinking Design Lens Challenge to Biology ©2015 Biomimicry 3.8. CC BY-NC-ND. Permission granted by Biomimicry 3.8 under Creative Commons.

**Figure 2:**
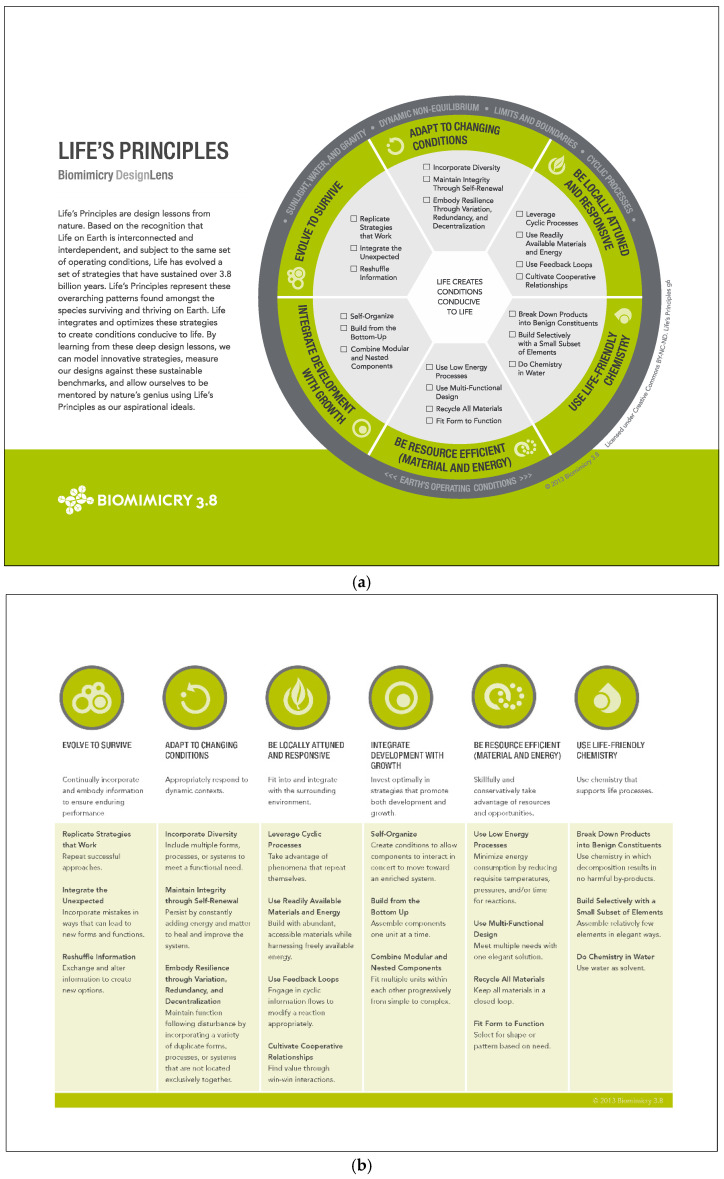
(**a**) Six main biomimicry life’s principles and their subprinciples. ©2015 Biomimicry 3.8. CC BY-NC-ND. Permission granted by Biomimicry 3.8 under Creative Commons. (**b**) Six main biomimicry life’s principles and their subprinciples. ©2015 Biomimicry 3.8. CC BY-NC-ND. Permission granted by Biomimicry 3.8 under Creative Commons.

An essential and integrated element of biomimicry thinking (Figure 1) are the LPs (Figure 2). LPs are overarching patterns in nature, typically employed in both the scoping and evaluation phases of the biomimicry design thinking process. They also offer an added set of inspiring directions to follow during the creation phase. LPs are the deep patterns of well-adapted design strategy lessons from nature, acting both as aspirational goals and sustainability benchmarks [17]. Integrating these strategies into human designs improves their function, resilience, and their potential to be regenerative. Patel and Mehta [18] describe LPs as the simple building blocks in nature that leverage interdependence within a constantly optimizing complex system. Kennedy [19] describes the use of LPs to identify unsustainable designs. 

The twenty-six LPs include twenty sub-principles that are clustered into six main principles each contributing to the comprehensive goal of ‘creating conditions conducive to life’. Each principle opens up pathways for seeking direct examples of model behavior. For example, if a design needs to adapt to changing conditions, the design team might look at the changing coat color of the arctic hare, white in the winter and brown in the summer, to see if a similar lesson might apply to their design’s contextual needs. Another example of an LP in a design is ‘build from the bottom up’ as observed in 3D printed products that use additive manufacturing, modular products, or User Experience, to create designs that are nested and easily shipped. Biomimicry practitioners can also use the LPs as an evaluation audit tool to check for missed opportunities for improving sustainability [17]. 

While biomimicry is a team effort, most biomimicry educators work alone. This article brings together four biomimicry educators who are all ASU MS Biomimicry graduates along with the director of the program. In an earlier research, the authors learned through a series of surveys and interviews [20] that learning the LPs influenced student thinking by increasing awareness of how integrating LPs contributes to design sustainability. Students have previously reported struggling with differentiating between and recalling all twenty-six LP subprinciples [21]. How can biomimicry educators improve their pedagogical practices to increase recognition, differentiation, and understanding of the LPs? How can biomimicry educators best prepare their students to integrate the LPs into their design thinking practice in order to create more sustainable human solutions? This article explores these questions.

In a previous manuscript, the authors conducted research on the translation between biology and design [22] that found dividing the Nature Technology Summary (NTS) exercise into sections with consecutive feedback loops, along with hand drawing of the mechanisms by students, improved the results. The addition of Life’s Principles (LPs) within these NTS exercises, was noted as helpful. The authors found that the integration of multiple LPs was desirable, leading to higher level systems-analogies, and increased life-centered design. 

In this manuscript, the authors reunite to evaluate the effectiveness of a novel LPs assignment by assessing the work of 110 students across three universities, Arizona State University (ASU) in Phoenix, Arizona (USA), College of Charleston (CofC) in Charleston, South Carolina (USA) and The Hague University of Applied Sciences (THUAS) in The Hague (The Netherlands). This introductory LP assignment allowed students to deeply explore, discuss and evaluate a single main LP and a sub LP in both biological and human design realms as an initial step in learning all of the LPs. In this study the authors assess our biomimicry students’ attempts to identify examples of LPs in nature and in human design.

## 2. Materials & Methods

Although biomimicry education is expanding, teachers and students still struggle with getting the science accurate and communicated visually into design principles that can be used for innovative ideas. The authors have the same background in biomimicry education, but teach at different schools to different student audiences. How can biomimicry educators rigorously funnel what they’ve learned through iterative curriculum development for such diverse audiences into recommended pedagogical principles? The overarching research question is: How can biomimicry educators improve their pedagogical practices to increase recognition and measure retention of nature’s overarching patterns, the ‘Life’s Principles’? Our sub-questions are:

- RQ 1: What elements of the LP exercise were students able to respond to with proficiency?
- RQ 2: What elements of the LP exercise did the students find challenging, and how might this assignment be iterated to improve student outcomes?
- RQ 3: What kind of potential did design students at THUAS and ASU see in the LPs as a tool for innovation and sustainability for their future designs?

In this study, the authors analyzed a single biomimicry LP assignment given across three separate university student cohorts in spring semester 2021. A quantitative and qualitative approach was used to improve result validity [23]. Student populations varied between undergraduate and graduate levels, ranging across a variety of disciplines. The disciplines of students included but were not limited to design, biology, architecture, entrepreneurship, etc. A total of 218 LP assignments created by 110 different students were evaluated (Table 1).

**Figure 3:**
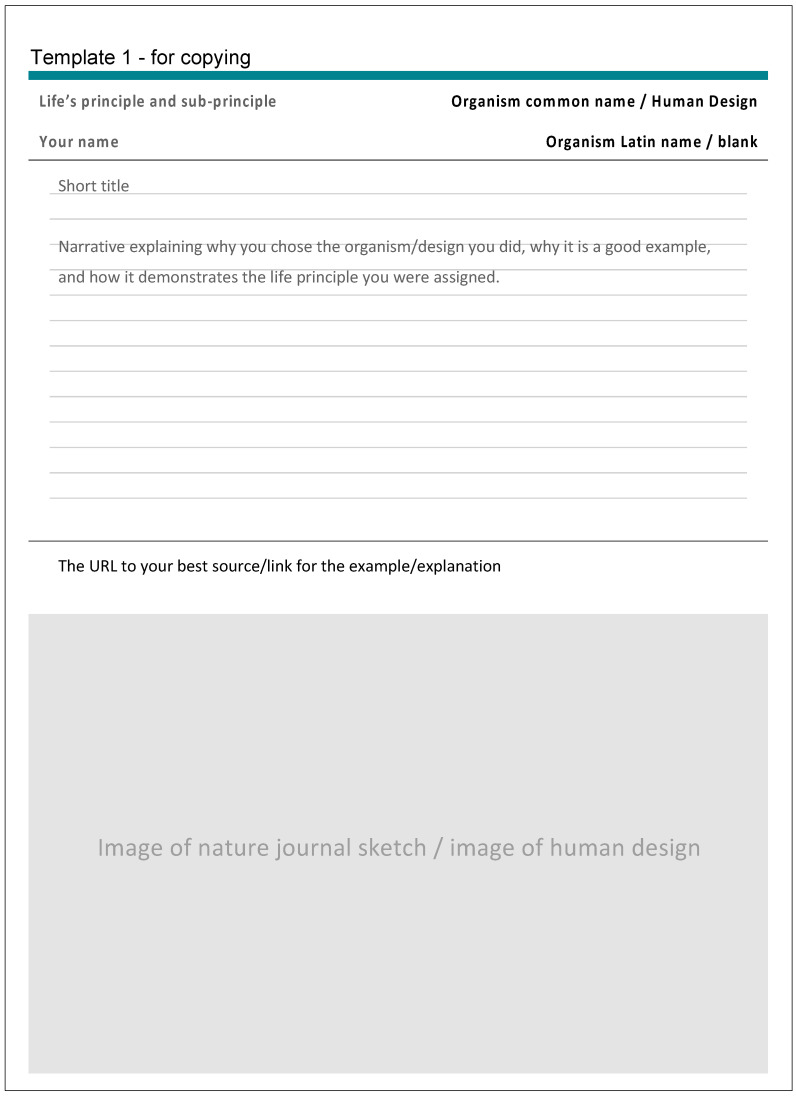
Exploring Life’s Principles in Nature and Design assignment template in google slides.

The students were introduced to a general overview of the six main LPs and then assigned LP related readings [17] and handouts (Figure 2a,b) by the authors. Students were then assigned to teams of 2–4 depending on class size. Each student was assigned 1–2 sub-LPs to research. A link to the Exploring Life’s Principles in Nature and Design assignment template Google Slides (Figure 3), was shared with all students. The template slides included:

- Student Name
- Name of Life’s Principle (Main and sub-principle)
- Name of the organism or design
- A short title of the organism or design example
- A written narrative about the example explaining why it is a good example of this specific LP
- The url link to the strongest source/resource for that example
- An image of both examples (design and biological in respective templates).

**Figure 4:**
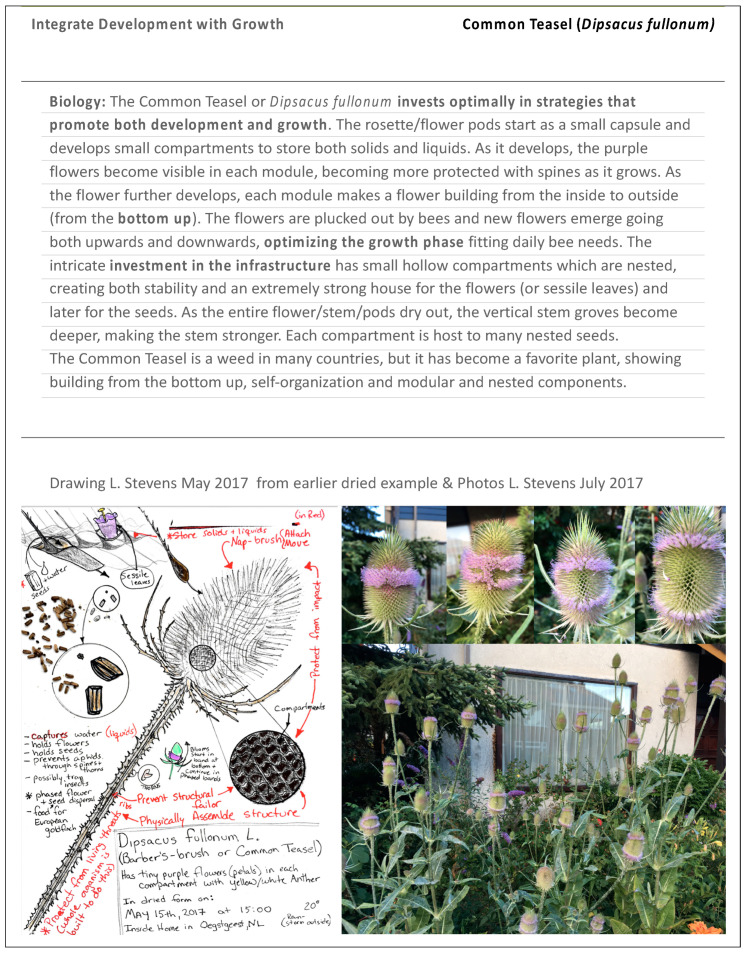
Example slide of LP in biology THUAS. Available online: https://www.nrcresearchpress.com/doi/pdfplus/10.4141/cjps75-122 (accessed on 26 January 2022).

**Figure 5:**
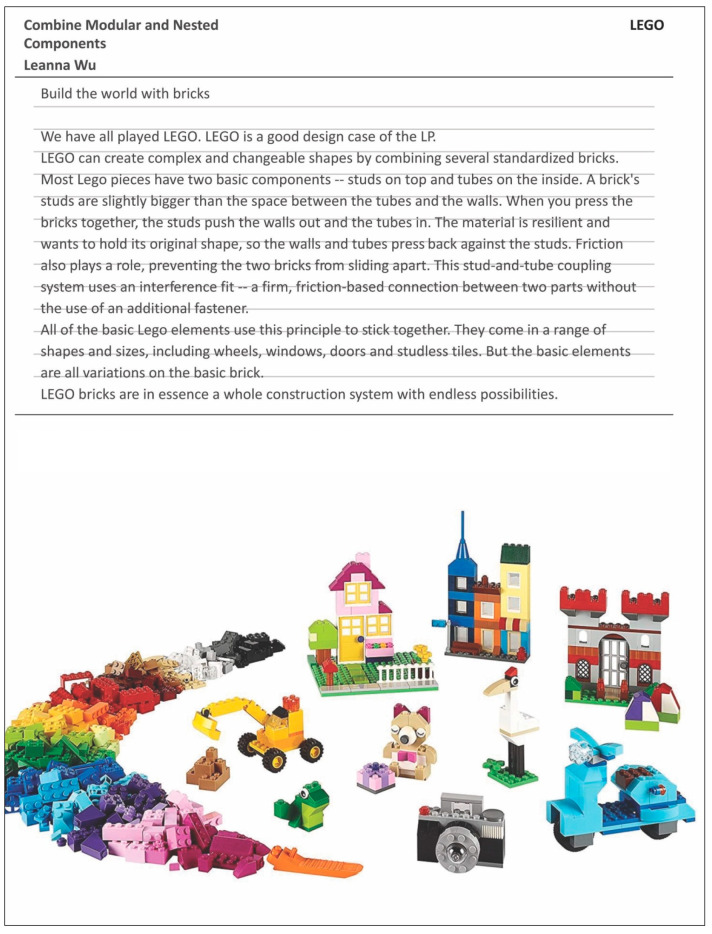
Example slide of LP in design ASU. Available online: https://entertainment.howstuffworks.com/lego.htm; https://www.amazon.com/LEGO-Classic-Largr-Creative-Brick/dp/B00NHQF6MG (accessed on 26 January 2022).

To help explain the assignment template slides, the authors shared examples of work done by previous students or by the faculty themselves. An example of the biological organism fitting the LP ‘Integrate Development with Growth’ is highlighted below (Figure 4) along with an example from a design fitting the LP ‘Combine Modular and Nested Components’ (Figure 5).

Students conducted research on their assigned LPs and individually completed the two slides in their template for the same LPs: one slide with a biological example and one with a human design example. Students were encouraged to go outside, search on Google Scholar and use biomimicry websites such as Ask Nature and Zygote Quarterly. Team members for identical main LPs shared and discussed their research over Teams or Zoom. ASU and CofC students added their team’s best examples to a ‘greatest hits’ slide deck. THUAS students discussed what their overarching LP meant.

**Figure 6:**
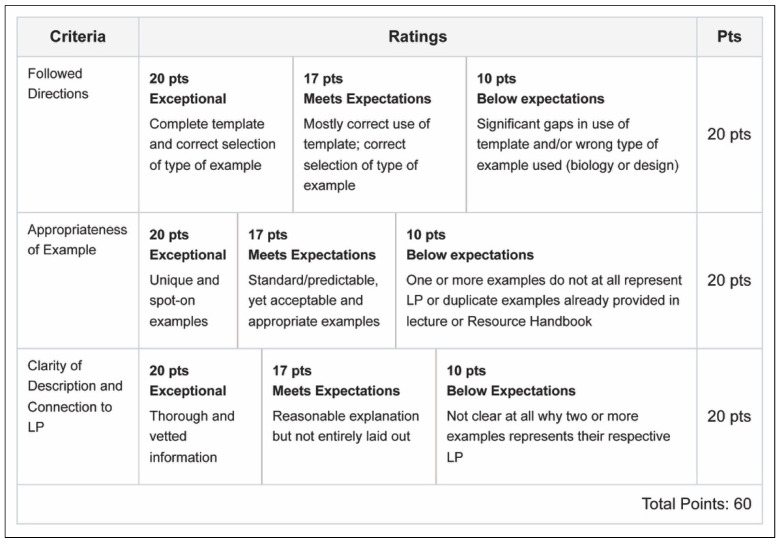
LP Rubric.

The authors identified pedagogical principles to create a common rubric (Figure 6). Student work was collected, anonymized, randomized, and shared in compliance with Institutional Review Board (IRB) approval and/or student consent for publication. Student assignments were scored by one external assessor using the common rubric. Criteria included following directions, appropriateness of LP examples, and clarity of description and connection to LP. Scores and reviewer comments were recorded in Google Sheets and exported to Microsoft Excel. Percentages of student work scoring proficient, acceptable, or unclear were calculated. Summary bar graphs and single factor analysis of variance (ANOVA) statistics were completed in Microsoft Excel. To test if any LP was more or less challenging for students than any other LP, single factor analysis of variance (ANOVA) statistical analyses were conducted. The authors tested for the effect of LP on student rubric scores. The null hypotheses tested were that there were no significant differences between the student rubric scores for following directions, providing suitable biological and human design examples, and clearly explaining their LP examples for each of the six main LP categories.

Although the authors used the same assignment, there were differences between student cohorts which are summarized below.

ASU

- Teams consisted of 6–8 randomly assigned students per main LP, resulting in 2 students per sub-principle each. No individual student was assigned to research a main LP. The insights about the main LP came from the team discussion and comparison at the end of the assignment during the assembly of the ‘best of’ slides.
- Students were asked to read the Life’s Principles Chapter in the Biomimicry Resource Handbook [17] and especially the section about their assigned LPs. At the end of the assignment, and before moving on to applying the LPs to their design project, they were asked to read about all the other LPs as well.
- The assignment encouraged students to go outdoors with their LP as a lens to find local organisms as much as possible. If this class was offered during a traditional semester, the class would have spent time outdoors together, but due to the virtual setting, it was not clear which students actually did go outside and which ones did most of their research online.

THUAS

- Teams consisted of 6–8 randomly assigned students per main LP, resulting in 2 students per sub-principle each, but were not asked to make a ‘best of’ slide deck as the last step of the exercise. Teams discussed the relevance of each sub-principle to decide on what elements are considered important for the main principle.
- THUAS students were given a second lecture during the introduction with more details about all 26 LPs during a separate class period.
- In their examples, THUAS students were asked to highlight in bold the factors that specifically fit the LP in order to visualize their reasoning.

CofC

- Teams consisted of 6–7 randomly assigned students per main LP. Students were also assigned to a sub-LP except for those assigned to the “Use Life Friendly Chemistry” LP, which was not subdivided.
- Students were tasked with finding biological examples that demonstrated their assigned LP while making independent outdoor nature observations using their assigned sub-LP as a search lens. They sketched their organism and explained why they chose it as an example of their assigned sub-LP, merging their LPs with an exercise called i-Sites (drawn observations in nature) to observe their organism [24].
- Students discussed their work as a team and chose ‘best of’ slides.
- The CofC class was hybrid with a face to face or Zoom option available to all students. Some students attended in person all semester long, some Zoomed all semester long, and some moved back and forth depending on health and fear concerns during the pandemic.
- The CofC class did not complete the exit survey due to course schedule and COVID-19 related constraints.
- ASU and THUAS design students completed a Google Forms exit survey (see Table 2) at the conclusion of the assignment, while CofC students did not undertake this survey. MAXQDA 2020 was utilized to analyze the survey response data, generate a word cloud, and create bar graphs.

## 3. Results

**Figure 7:**
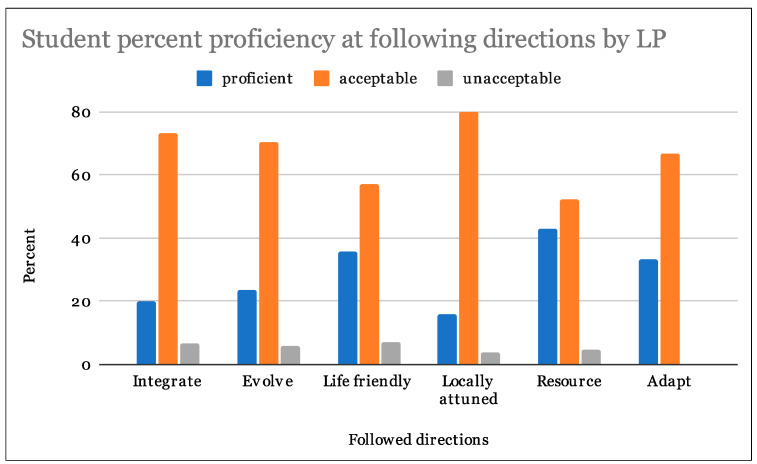
Student proficiency at following directions by LP.

**Figure 8:**
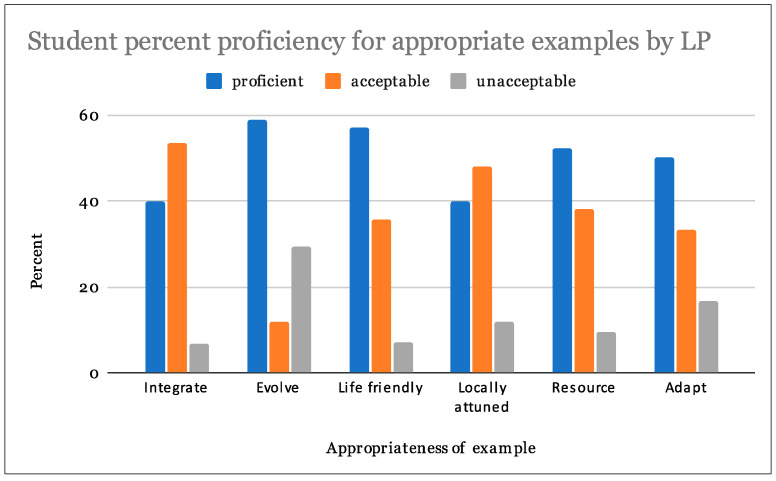
Student proficiency at choosing appropriate examples by LP.

**Figure 9:**
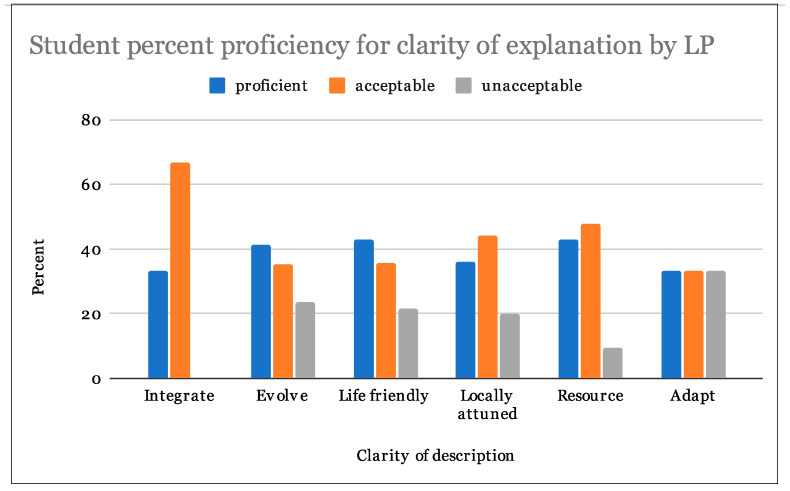
Student proficiency for clarity of explanation by LP.

From our analysis, 28% of students were able to follow directions at a proficient level, and 67% at an acceptable level. In regards to the appropriateness of examples of LPs in biological and human systems, 49% percent of students scored proficient while 37% were acceptable. Assigned LP examples were also evaluated on the basis of clarity, 38% students scored proficient and 44% were acceptable (Table 3). Students achieved the highest proficiency scores in their ability to find appropriate examples of the LPs (Figure 7, Figure 8 and Figure 9). 

Single factor analysis of variance (ANOVA) statistical analyses tested for the effect of LP on student rubric scores. Results indicate a failure to reject the null hypotheses in all cases (*p* > 0.05). There were no significant differences in student rubric scores for assigned LPs for following directions (Table 4, *p* = 0.50), providing suitable biological and human design examples (Table 5, *p* = 0.89) or clearly explaining their LP examples (Table 6, *p* = 0.59). Students assigned any particular LP did not perform any better or worse than students assigned any other LP. Please see table legend for Table 4, Table 5 and Table 6 for explanation of table abbreviations.

**LP legend for Table 3, Table 4, Table 5 and Table 6:** LP-Adapt: Adapt to Changing Conditions; LP-Integrate: Integrate Development with Growth; LP-Evolve: Evolve to Survive; LP-Life: Use Life-friendly Chemistry; LP-Local: Be Locally Attuned and Responsive; LP-Resource: Be Resource Efficient (Material and Energy).

**Legend for Table 4, Table 5 and Table 6:** Groups: assigned LPs; LP-Adapt: Adapt to Changing Conditions, LP-Integrate: Integrate Development with Growth, LP-Evolve: Evolve to Survive, LP-Life: Use Life-Friendly Chemistry, LP-Local: Be Locally Attuned and Responsive, LP-Resource: Be Resource Efficient (Material and Energy), Count: number of students per group, Sum: Sum of student scores, Average: mean student score, Variance: variance of student scores, SS: Sum of squares, df: Degrees of freedom, MS: Mean square, F: F statistic, *P* value: Probability, F crit: Critical value of F.

**Figure 10:**
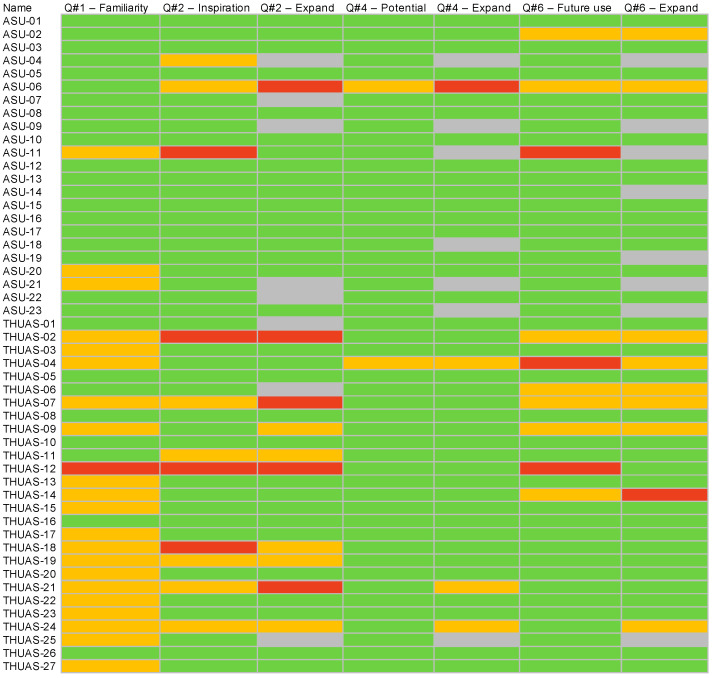
Individual student exit survey data showing student response ranks (Q1, 2, 4, 6) of 4–5 = positive (green), 3 = ambivalent (orange), and 1–2 = negative (red) survey responses and MAXQDA coded open responses (Q3, 5, 7) as positive (green), ambivalent (orange) and negative (red). Grey indicates no or inapplicable answers.

**Figure 11:**
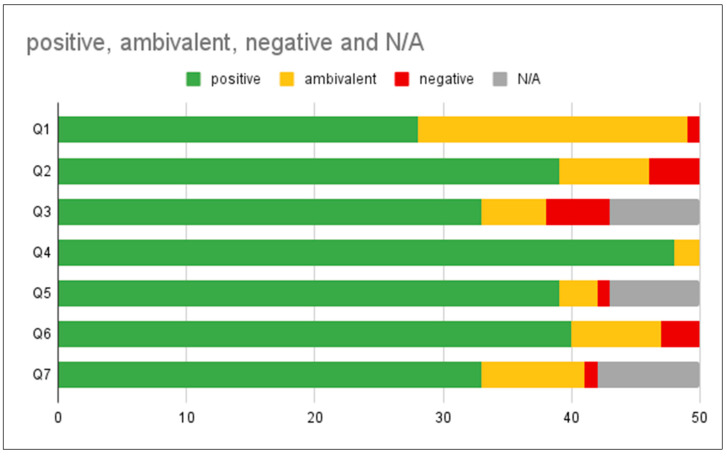
Showing summary results of exit survey Q1–7. (see Table 3 for exit survey questions) Data ranks (Q1, 2, 4, 6) were scored as 4–5 = positive (green), 3 = ambivalent (orange), 1–2 = negative (red); open responses (Q3, 5, 7) were coded positive (green) ambivalent (orange) and negative (red). Gray indicates no or inapplicable answers.

The THUAS and ASU exit survey responses (n = 50) indicate that every student made positive comments overall (Figure 10 and Figure 11). A total of 26 students made ambivalent comments and 9 students made negative comments in the survey free response questions (Q3, 5, 7) (Figure 10).

**Figure 12:**
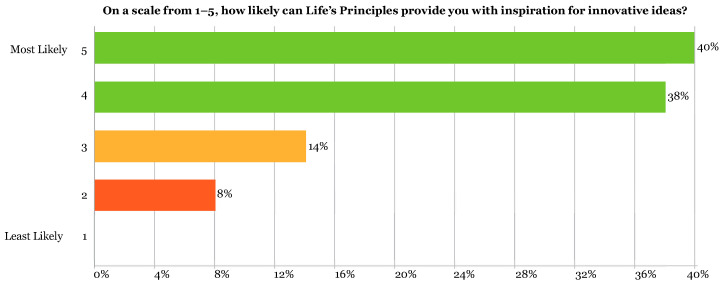
Showing results of exit survey Q2: On a scale from 1–5, how likely can LPs provide you with inspiration for innovative ideas? Data were scored as 4–5 = positive (green), 3 = ambivalent (orange), 1–2 = negative (red).

**Figure 13:**
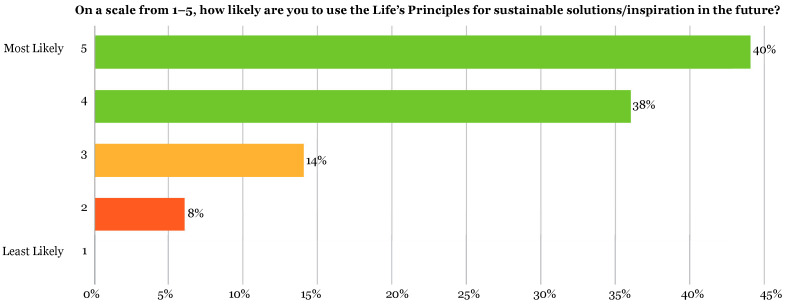
Showing results of exit survey Q6: On a scale of 1–5, how likely are you to use the LPs for sustainable solutions/inspiration in the future? Data were scored as 4–5 = positive (green), 3 = ambivalent (orange), 1–2 = negative (red).

Survey answers of 39 students, or 78% of respondents (40% = 5, 38% = 4) saw potential in getting inspiration for innovative ideas from the LPs. Only 11 students (22%) (14% = 3, 8% = 2) were ambivalent about whether the LPs could be a tool for innovative design (Figure 12). These responses all came from the negative survey answers from a total of 9 students (red boxes, Figure 10). These concerns aligned with the answers revealing students’ lack of confidence to apply them correctly. In the survey responses, 40 students (80%) indicated that they would be likely to use the LPs as part of their design process in the future while 22 students (44%) leaned towards highly likely (Figure 13).

**Figure 14:**
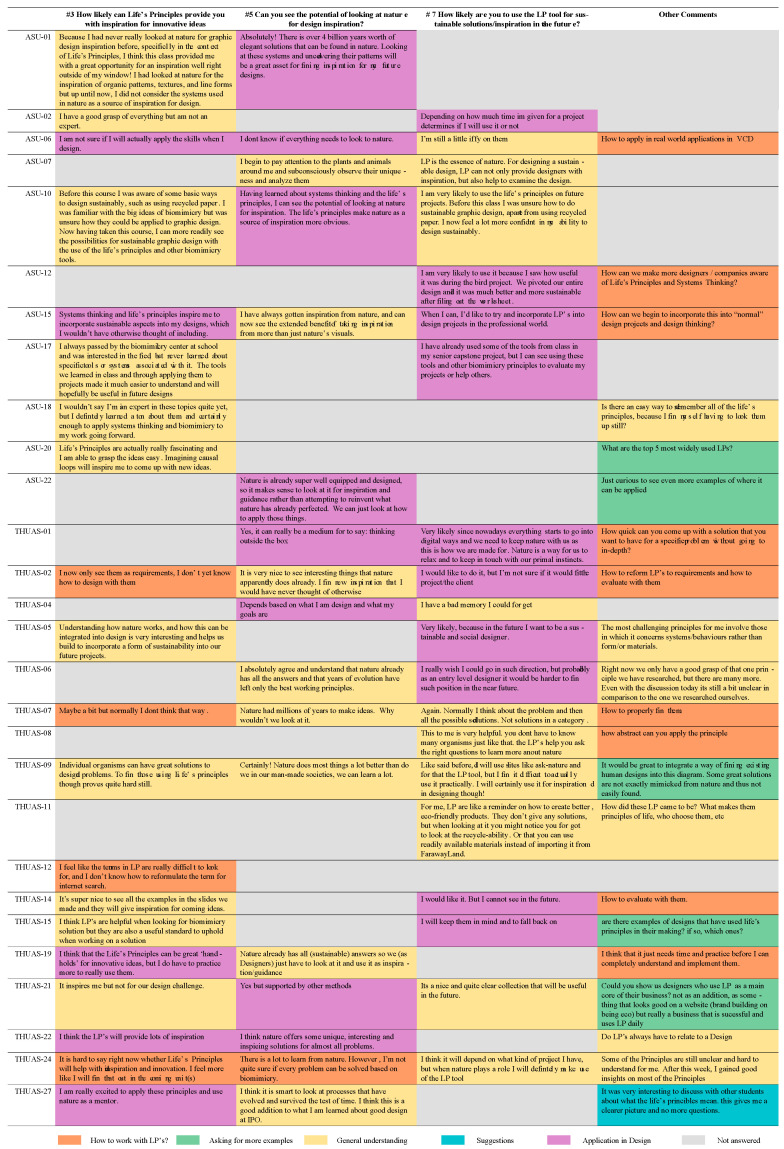
Collection of some of the most insightful responses from the survey. Color legend categorizes comments. Gray boxes indicate no or inapplicable responses.

Free response exit survey comments were categorized and visually represented in Figure 14 below. Multiple respondents voiced the need for more practice with the LPs. Eight students (16%) mentioned that they find the LPs a bit hard to understand well enough to apply them correctly. While one student explicitly asked if there is a trick on how to memorize them, another student also highlighted the struggle with understanding the systems-based LPs since doing so is more complex than understanding form or material LPs. From the survey, it was evident that 7 students were unclear on the applicability of the LPs, highlighting that they are unsure or unwilling to apply the LPs into their design process in the future. One student said: “I don’t know if everything needs to look to nature” ~ASU-06. Nine respondents (18%) indicated that although the LPs can be inspiring, they failed to see the potential for LPs to be included in the design process (Figure 14). 

**Figure 15:**
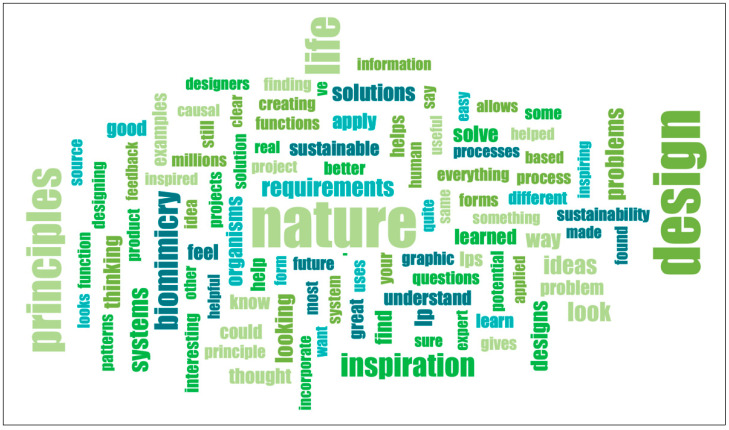
Word cloud of combined free response answers in survey (Q3, 5, 7). Generated in MAXQDA2020.

A word cloud (Figure 15) of the answers to the open-ended questions revealed that the word “Nature” was 1st, “Design” 2nd, “Biomimicry” 3rd, and “Inspiration” was ranked 4th place. The words “Life” and “Principles” were eliminated from this ranking because they mention the name of the assignment itself.

## 4. Discussion

The authors acknowledge that there is a possibility that impact bias could have influenced the survey responses. Impact bias has been studied in student evaluative responses. It describes the overestimation of how positive or negative the students’ feelings are about a specific experience [25]. Through another survey or interviews at the end of the semester during future studies the authors can find changes in student perception of the assignment over a longer period of time. 

### 4.1. RQ 1: What Elements of the LP Exercise Were Students Able to Respond to with Proficiency?

The assignment directions were effective and 95% of the students followed them at an acceptable or proficient level. When explaining the chosen organism or design fitting their sub-principle, 85% of students submitted proficient or acceptable work and 82% did so with clarity (Table 2). ANOVA results indicate that no LP was any more challenging for students to work with than any other LP (Table 4, Table 5 and Table 6). The authors see that most students who found appropriate examples also gave clear descriptions and reasoning why their examples fit the principle. Yen et al. [9] noted that students struggled to explain their analogical reasoning when bridging biological and human engineering and that this was exacerbated by the breadth and number of biological systems with which students were working. The elegant simplicity of the LPs may assist students with making stronger analogies. Requiring THUAS students to highlight the signal terms of the sub-principle in each example may have helped students self-evaluate whether their found model is a good example of the LP. The majority of students who scored low, scored as such across the rubric. The authors used the rubric to identify improvements in the course [26] and in collective biomimicry education programs. The authors agree with [26,27] that the rubric can be improved on the following three elements to articulate expectations: (1) evaluation criteria; (2) quality definitions; and (3) a scoring strategy. To do so, it is essential to include and explain the grading rubric to participants, be more specific in highlighting the essential key terms from the given literature, and explain the importance of the scoring categories. Furthermore, providing visual examples throughout the process can help students evaluate what is relevant for the translation of biology to human systems [10]. While this exercise was carried out in an online/hybrid setting during the COVID-19 pandemic, it is likely that doing this exercise in a full face-to-face context with a physical instructor present might result in deeper participation across the board [28]. The possibility of having multi-sensory iterative feedback loops from the instructor and the ability of students to share what in real-time, would likely deepen their acquired knowledge [29]. However, the results of this introductory exercise indicate that students were able to understand and find examples fitting each LP. 

### 4.2. RQ 2: What Elements of the LP Exercise Did the Students Find Challenging, and How Might This Assignment Be Iterated to Improve Student Outcomes?

Most of the ambivalent or negative student exit survey responses were comments that described the difficulty of remembering the LPs or indicated that students did not yet see how the LPs could be applied to the design process. One of the challenges mentioned by the students was the need to find ways to memorize the LPs in order to improve confidence in working with them. Student exit survey responses indicated some challenges with understanding the complexity behind the system that nature operates within. A few also wondered how the LPs can be applied in the design process. One factor to consider is that the context changes for each design problem and thus memorizing the LP might not be a worthwhile undertaking, but rather the application of and an evaluation concerning this change would yield greater impact [7].

A large percentage of students indicated that they wanted more practice with the LPs. Since this assignment introduced them to only one of the LPs, they felt a lack of comparable knowledge about all of the LPs. “Right now we only have a good grasp of that one principle we have researched, but there are many more. Even with the discussion today it’s still a bit unclear in comparison to the one we researched ourselves” ~THUAS-06. The authors acknowledge the challenge of time vs content in any course. One way to overcome this is to introduce the LPs early on in a class, and then continually and repeatedly integrate them in subsequent assignments. The reiteration and continued application of the LPs will provide a bit more experience, understanding, and retention of all the LPs [30]. Furthermore, introducing the LPs through active learning has shown to increase student understanding [31]. Active learning methods for the LPs could include hands-on activities with natural artifacts, or immersive outdoor explorations with a lens on particular LPs similar to what CofC did with the iSites (see Description of Common Assignment). Nonetheless, the LPs take up a full-semester advanced course in the Biomimicry Master’s program at ASU, so it is unrealistic to expect the students to get deep knowledge of all of the 26 LPs during an introductory level assignment. 

Respondent comments also hinted at the difficulty of seeing how to work with LPs and how they are applied in a design process (“How to work with LP’s” orange boxes in Figure 14). This study simply investigated the immediate knowledge gained from one activity. Some students commented that they cannot yet answer whether they see the potential of applying the LPs in their future projects. In some classes, the LPs became part of a design process following this particular assignment. After having completed the entire design process, students would have gained more insights into how the LPs guided their decisions. A second survey at the end of the semester could be worthwhile to see if some of the applications of the LPs helped make it more clear for the students. 

Including activities that allow students to experience how the LPs can be incorporated into the design process will also help reduce the confusion of application in design (purple boxes Figure 14). Furthermore, many comments from the survey asked for more examples (green boxes, Figure 14). A best practice in bio-inspired design education identified by [10] is the exposure to a breadth of examples in nature. Studies have also shown that providing examples from previous cohorts increases effectiveness of an assignment [32]. Therefore, including examples of how the LPs have already been applied during the introductory phase of this assignment could help strengthen the context and the reasoning for learning the LPs in the first place.

In the category of general understanding (yellow box, Figure 14), students commented that they wanted to have all the LPs memorized by the end of this exercise. “Is there an easy way to remember all of the LPs, because I find myself having to look them up still?” ~ASU-18. The authors have been working with the LPs for quite a few years, and still tend to use the reference sheet. They are also considering the design of a mnemonic teaching device for the LPs as a collaborative next step. Moving forward, the assignment and introduction of the LPs will have to set the realistic expectation clearly that the goal is not to learn these LPs to the extent of memorizing them completely. Understanding the nuances of each LP, as well as having ideas on how they can be applied is the goal. 

The CofC, THUAS, and ASU students were asked to discuss their found LP examples by meeting in teams per main LP. In those discussions, ASU and CofC students also compared their examples and chose one as the ‘best of’ example for that sub-LP. Comments in the survey revealed that the ASU students felt that step was very helpful. Even though the classes were mostly virtual, the exchange and reflection that happened during those discussions helped students refine their knowledge about the LPs (blue box, Figure 14). Word cloud results (Figure 15) showed repeated positive key words which can be an indicator that students feel the LPs provide a good tool for design inspirations in their work.

A rewarding discovery was that students seemed to recognize and learn about the complexity of nature through this assignment. Even if they did not fully understand it or feel confident in identifying the nuances of systems, the fact that design students learned that nature is a complex system was a win for the assignment. “The most challenging principles for me involve those in which it concerns systems/behaviors rather than form/or materials” ~THUAS-05. Natural systems are diverse and intricately interconnected. Many LPs describe this non-linear relationship. The authors feel that including some activities around systems thinking in the semester could help in understanding those LPs on a deeper level [33]. 

### 4.3. RQ 3: What Kind of Potential Did Design Students at THUAS and ASU See in the LPs as a Tool for Innovation and Sustainability for Their Future Designs?

Through this research study the authors learned that by doing the Exploring Life’s Principles in Nature and Design assignment slide exercise, the students were introduced to the LPs, but this did not necessarily mean that they understood the LPs well enough to apply them in a design. Furthermore, the level to which they learned how LPs are integrated within a solution cannot be accurately measured until they apply them during a design process. This exercise was not evaluating a final level of knowledge, but an initial iteration of the principles to later embed these into their design process. 

The survey confirmed that the assignment helped the students get introduced to the LPs. In addition, almost all students felt there was great potential in the LPs and nature itself for innovation and sustainability but were unsure how to apply them. It is clear that they understood enough from this assignment about the LPs to know that they could be used as inspiration (Figure 13), where 80% of students selected 4 or 5, selecting “positive” to the question of innovation potential. Conducting a future study on the impact the LPs had on their design decisions can be done with future cohorts to determine how this impacts the innovation and sustainability of their design solutions.

## 5. Conclusions

The authors’ research aimed to discover how to improve their pedagogical practices to increase recognition and retention of nature’s overarching patterns, the ‘LPs’. The authors also aimed to uncover common misconceptions and look for factors to improve our measuring rubric and the template exercise, adding suggestions and comments from students after completion. As this exercise was an introduction to LPs, the authors found that many students were able to retain the set of guidelines when adding these directly into their template. In future iterations, the authors shall point out adding key words from the LP guidelines (Figure 2b) to clearly guide students while learning the principles for the first time. Students requested to see more than just the one example of each LP that the authors provided, and requested a clear explanation of why these were meaningful examples. While each instructor felt that these requests were already fulfilled, perhaps an iterative explanation of more example organisms and designs is needed. With the many proficient examples made by this cohort, the authors are empowered to expand the exercise in this manner in future courses. Repeated LP exercises, more nuanced explanations, active learning, more examples, and a rubric specifying use of key words and sentence cues should improve student understanding.

*“I believe design comes with love; I believe design is more than aesthetics; I believe good design is easy to understand and to apply in life; I believe design can make a real difference in life. The LPs give the idea about “how”. I will keep learning, and bring more sustainability into my design”*

ASU-23

## Figures and Tables

**Table 1:** Research context, cohort participants.

Institution	The Hague University of Applied Sciences (THUAS)	Arizona State University (ASU)	College of Charleston (CofC)
Location	The Hague, NL	Tempe, Arizona, USA	Charleston, South Carolina, USA
Audience	Design, Engineering, other miscellaneous technical fields	Architecture, Industrial Design, Interior Arch., Visual Communication Design	Biology, Entrepreneurship, Urban Studies, Environmental and Sustainability Science
Level	Undergraduate	Undergraduate & Graduate	Undergraduate
Cohort dates:	Spring 2021	Spring 2021	Spring 2021
Number of participants	*n* = 37	*n* = 36	*n* = 37
Student Background	Minor for exchange students (motivation letter) or 4th semester for Industrial Design Engineering students	Undergraduate and Graduate students from various design disciplines (Architecture, Interior Architecture, Industrial Design, Visual Communication Design)	Variable. Upper level undergraduate. No prior design experience.
Course name(s)	Design with Nature, Industrial Design Engineering semester	Sustainable Graphic Design	Special Topics: Biomimicry Thinking

**Table 2:** Survey questions numbered for reference.

Question #	
1	On a scale of 1–5, please indicate how familiar you feel you are now with the Life’s Principles in Design
2	On a scale of 1–5, how likely can Life’s Principles provide you with inspiration for innovative ideas?
3	Please expand on your answers above
4	Can you see the potential of looking at nature for design inspiration?
5	Please expand on your answer above
6	On a scale of 1–5, how likely are you to use the LPs for sustainable solutions/inspiration in the future?
7	Please expand on your answer above

**Table 3:** Life’s Principles rubric data collection summary.

Rubric	LP-Adapt	LP-Integrate	LP-Evolve	LP-Life	LP-Local	LP-Resource	Total	%	Level
Followed Directions	6	3	4	5	4	9	31	28%	Proficient
	12	11	12	8	20	11	74	67%	Acceptable
	0	1	1	1	1	1	5	5%	Unclear
Total	18	15	17	14	25	21	110	100%	
Appropriate Examples	9	6	10	8	10	11	54	49%	Proficient
	6	8	2	5	12	8	41	37%	Acceptable
	3	1	5	1	3	2	15	14%	Unclear
Total	18	15	17	14	25	21	110	100%	
Clarity	6	5	7	6	9	9	42	38%	Proficient
	6	10	6	5	11	10	48	44%	Acceptable
	6	0	4	3	5	2	20	18%	Unclear
Total	18	15	17	14	25	21	110	100%	

**Table 4:** Summary table single factor ANOVA for followed directions.

SUMMARY						
Groups	Count	Sum	Average	Variance		
LP-Adapt	18	24	1.34	0.24		
LP-Integrate	15	17	1.14	0.27		
LP-Evolve	17	20	1.18	0.28		
LP-Life	14	18	1.29	0.37		
LP-Local	25	28	1.12	0.19		
LP-Resource	21	29	1.38	0.35		
ANOVA						
Source of Variation	SS	df	MS	F	*p*-value	F crit
Between Groups	1.2	5	0.24	0.87	0.50	2.30
Within Groups	28.65	104	0.28			
Total	29.85	109				

**Table 5:** Summary table one way ANOVA for appropriateness.

SUMMARY						
Groups	Count	Sum	Average	Variance		
LP-Adapt	18	53	2.94	1.61		
LP-Integrate	15	44.5	2.97	1.20		
LP-Evolve	17	45	2.65	3.34		
LP-Life	13	42	3.23	1.44		
LP-Local	25	72.5	2.90	1.29		
LP-Resource	21	64	3.05	1.55		
ANOVA						
Source of Variation	SS	df	MS	F	*p*-value	F crit
Between Groups	2.85	5	0.57	0.33	0.89	2.30
Within Groups	176.82	103	1.72			
Total	179.67	108				

**Table 6:** Summary table one way ANOVA for clarity.

SUMMARY						
Groups	Count	Sum	Average	Variance		
LP-Adapt	18	40	2.22	2.68		
LP-Integrate	15	45.5	3.03	0.52		
LP-Evolve	17	43	2.53	2.55		
LP-Life	14	36.5	2.60	2.47		
LP-Local	25	63.5	2.54	2.14		
LP-Resource	21	61	2.90	1.47		
ANOVA						
Source of Variation	SS	df	MS	F	*p*-value	F crit
Between Groups	7.40	5	1.48	0.75	0.59	2.30
Within Groups	206.44	104	1.98			
Total	213.84	109				

## Data Availability

All the raw data is stored on a google drive behind a two-factor authentication wall by the participating universities.
